# Preterm Infant Feeding: A Mechanistic Comparison between a Vacuum Triggered Novel Teat and Breastfeeding

**DOI:** 10.3390/nu10030376

**Published:** 2018-03-19

**Authors:** Donna Geddes, Chooi Kok, Kathryn Nancarrow, Anna Hepworth, Karen Simmer

**Affiliations:** 1 School of Molecular Sciences, Faculty of Science, The University of Western Australia, Perth, WA 6009, Australia; arhepworth@westnet.com.au; 2 Centre for Neonatal Research and Education, School of Medicine, Faculty of Health and Medical Sciences, The University of Western Australia, Perth, WA 6009, Australia; Chooi.Kok@health.wa.gov.au (C.K.); Nancarrow.Kathryn@health.wa.gov.au (K.N.); Karen.Simmer@health.wa.gov.au (K.S.); 3 The Neonatal Directorate, King Edward Memorial Hospital, Perth, WA 6008, Australia

**Keywords:** infant, feeding, preterm, premature, bottle, human milk, breastfeeding, nipple shield, infant feeding

## Abstract

The goal for preterm infants is to achieve full oral feeds quickly and ultimately progress to full breastfeeding. Supplementary oral feeds are often given when the mother is not available to breastfeed. Bottles typically deliver milk in a different fashion compared to breastfeeding, which is thought to hamper transition to full breastfeeding. The aim of this study was to compare the sucking dynamics of preterm infants fed at the breast to feeding with an experimental novel teat (NT) designed to release milk only upon the application of vacuum. Simultaneous ultrasound imaging of the infant oral cavity and measurement of intra-oral vacuum was performed during a breastfeed and a feed with the NT. Test weighs were used to measure milk intake. Linear mixed effects models were performed to investigate differences by feed type, and simultaneous linear regression was performed to investigate individual patterns. Tongue movement was not different between breastfeeding and the NT. Intra-oral vacuums (median (interquartile range: IQR)) were significantly lower with the NT (Baseline vacuum: −5.8 mmHg (−11.0, 0.1); Peak: 40.0 mmHg (−54.6, −27.1)) compared to breastfeeding (Baseline: −31.1 mmHg (−60.0, −12.7); Peak: −106.2 mmHg (−153.0, −65.5)). Milk intake was significantly higher with the NT (33 mL (22.5, 42.5)) compared to the breastfeed (12 mL (3, 15.5)). The novel teat encouraged a similar tongue action to breastfeeding, and infants transferred a greater volume of milk with the novel teat. Intra-oral vacuums were lower in strength with the novel teat compared to the breast. Use of the novel teat for the training of sucking dynamics in preterm infants has the potential to improve breastfeeding success and requires further investigation.

## 1. Introduction

The importance of human milk (HM) for the preterm infant cannot be understated. In addition to protecting the infant from infection, which can often be life threatening for these infants, and providing nutrients for growth, several studies have linked HM with improved long-term health outcomes. Most recently, donor HM–fed preterm infants have been shown to have better cardiac morphology in adulthood compared to those fed high protein formulas, and these differences potentially increase the likelihood of better cardiopulmonary outcomes later in life [1]. Further, improved neurodevelopment [2,3] later in life has been documented in this population despite predominantly HM-fed preterm infants having slower growth rates than those that are formula-fed [4]. Predominant HM feeds in the first 28 days of life has been linked to a greater volume of deep gray matter volume at term along with higher IQ, working memory, academic achievement, and motor function at seven years of age in infants born less than 30 weeks gestation [5]. 

These important benefits of HM often overshadow the development of the oro-facial structures due to the necessity for provision of adequate infant nutrition. Preterm infant nutrition is a tenuous balance of type of nutrition, fortification, mode of delivery, and volume of feeds the infant can tolerate. As such, the mechanics of feeding or the delivery of milk are unfortunately largely underemphasized. The bones of the skull and face are malleable in early life, and stresses or lack of stress impacts bone growth [6]. Indeed, there are suggestions that extrinsic factors such as changes in head shape due to infant position and orotracheal intubation [7,8] can alter both palate size and shape, with the potential to affect the entire nasomaxillary complex, particularly in the preterm infant. Further, intrinsic factors such as under nutrition, endocrine anomalies, central nervous system impairments, metabolic dysfunction, and particular medications may impact development of the nasomaxillary complex [9].

Thus, the positive associations of HM and breastfeeding with both short- and long-term health outcomes as well as better development of infant structure and form [10] underpins the goal of the Neonatal Intensive Care Unit (NICU) to facilitate full breastfeeding. Unfortunately, achievement of this goal is hampered by the immaturity of the infant as well as infant co-morbidities such as bronchopulmonary dysplasia, gastrointestinal surgeries, sepsis, neurological impairment, and growth [11], which is further compounded by a range of feed competence between infants [12]. Since discharge of the infant home is also a major goal of the NICU due to evidence suggesting preterm infant health is improved [13], achievement of full oral feeds that are both safe and efficient is imperative. Independent oral feeding is a requirement for discharge home, and often, this may be the last milestone the infant met prior to discharge [14]. More rapid discharge can be achieved by supplementing infants with expressed HM milk via a bottle whilst breastfeeding is being established [12], and this strategy is commonly employed in many units due to infant health, availability of the mother to breastfeed, and economic constraints. 

In the pursuit of full breastfeeding in the preterm infant, one must recognise that marked differences in the delivery of milk exist between breastfeeding and bottle-feeding. During bottle-feeding, milk is constantly available to the infant. However, during breastfeeding, milk is only available at milk ejection, and during this transient period, milk flow rates vary markedly. Milk flow rates are important as preterm infants may not be mature enough to regulate milk flow and co-ordinate sucking, swallowing, and breathing at the same time [15]. High flow rates require the infant to swallow frequently to clear the milk from the oral cavity and increase the risk of aspiration [16]. A bottle designed for term infants to release milk only when the infant sucks (applies vacuum; Calma, Medela AG, Baar, Switzerland) has been shown to have a similar tongue action, suck-swallow-breathe patterns, and equivalent heart rate and oxygen saturation to breastfeeding [17]. It was speculated that a similar bottle design for preterm infants would encourage a similar tongue action to breastfeeding when intra-oral vacuum was applied.

The aim of this study was to compare the sucking dynamics of preterm infants fed at the breast and with an experimental novel teat (NT) designed to release milk only upon the application of vacuum and stimulate a tongue movement similar to that of breast feeding.

## 2. Materials and Methods

### 2.1. Participants

A convenience sample of 17 mothers and infants (birth gestation age: 23.6–33.3 weeks; post menstrual age (PMA) 32.7–39.9 weeks) whose mothers intended to breastfeed were examined in the special care nurseries of King Edward Memorial Hospital for Women (KEMH), Perth between 1 August 2011 and 30 June 2012. These participants were part of a randomized controlled trial [18] to assess the efficacy of a novel teat designed compared to a conventional teat to meet the needs of the developing preterm infant (Australian New Zealand Clinical Trials Registry, ACTRN12614000875606 [19]).

Inclusion criteria were infants of gestational age (GA) 25 to 34 weeks whose mothers intended to breastfeed and who required 75% enteral feeds by intragastric tube with the remainder provided by intravenous fluids. Exclusion criteria were congenital anomalies, grade 4 intra-cerebral hemorrhage, and periventricular leukomalacia and oral anomalies (for example, ankyloglossia, cleft palate) [18]. 

The study period was from 1 August 2011 to 30 June 2012. Breastfeeds were timed when the mother was available to feed and the infant could attach to the breast. 15/17 infants therefore were monitored during a breastfeed prior to the NT as it is policy not to introduce bottle feeds until after an infant has demonstrated the ability to attach and suckle at the breast. Monitored sessions were one day apart for eight infants and the same day for two infants, with the remainder ranging between two and seven days. Infant sucking dynamics has previously been shown to be similar to feeding without a nipple shield in a cross-sectional study [20].

For this sub analysis, due to limited resources, only the NT was assessed to determine if the incorporated features encouraged a suck mechanism was similar to breastfeeding. As per standard clinical practice of the study hospital, a nipple shield was used for all breastfeeds where the infant was unable to sustain attachment at the breast. Typically, mothers regularly attempted attachment without a nipple shield and proceeded with a nipple shield if the infant was unable to achieve and/or sustain attachment. 

Exclusion criteria included oro-facial anomalies with the potential to affect feeding, grade IV intra-cranial haemorrhage, and other congenital anomalies. All infants were latching and sucking at the breast before participating. 

Mothers supplied written, informed consent to participate in the study, which was approved by the Scientific Research Ethics Committee of King Edward Memorial Hospital.

### 2.2. Experimental Novel Teat

The infants were fed with a novel teat (Medela AG, Baar, Switzerland) that was designed to integrate oral feeding skills known to improve outcomes: development of vacuum [21] and self-paced feeding [22]. A shut-off valve ensured milk flowed only when the infant created a vacuum, and venting ensured the NT did not collapse. All infants fed with the valve threshold level of −10 ± 5 mmHg [18].

### 2.3. Ultrasound Imaging and Measurement of Intra-Oral Pressure

Submental ultrasound scans of the midline of the infant’s oral cavity as described previously [23,24,25] were acquired with a Sonsite TITAN system and an endocavity convex transducer ICT 8-5 MHz (SonoSite Inc., Bothell, WA, USA). Infant intra-oral pressures were measured via a small Silastic tube filled with sterile water and taped alongside the nipple and attached to a disposable pressure transducer (Cobe Laboratories, Frenchs Forest, NSW, Australia). The transducer was connected to the amp bridge (ADInstruments, Castle Hill, NSW, Australia), and the output was recorded using MacLab (ADInstruments) and software package Chart v5.0.2 (ADInstruments) on a laptop computer (Mac OS X v10.3.8, Apple Inc., Kupatino, CA, USA). Signals from the ultrasound machine and pressure transducer were recorded simultaneously with a Video Capture Module (ADInstruments) for the entire feed. 

### 2.4. Ultrasound and Intra-Oral Vacuum Measurement

The first three well-visualized nutritive suck cycles were selected from each feed. Tongue and nipple movement were measured from two images of each suck cycle when the mid tongue was at its highest (TU) and lowest point (TD) [26] using Screen Calipers v 3.2 (Iconico Inc., New York, NY, USA). Measurements made included nipple to hard–soft palate junction (N-HSPJ), intra-oral depth (IOD; vertical measurement of the mid tongue lowering creating the space accommodating the milk bolus), and nipple diameter at 2, 5, 10, 15, and 20 mm from the tip of the nipple. 

Measures of suck bursts included mean minimum pressure (peak vacuum) and mean maximum pressure (baseline vacuum), mean pressure, maximum and minimum pressure, suck rate, and duration of the burst. Mean pressure and pause duration were also measured. 

### 2.5. Infant Milk Intake

Milk intake was measured by test weights made before and after the breastfeed [27] (Baby Weigh Scale, Medela AG, Baar, Switzerland). Milk transfer (mL/min) was calculated by dividing the volume of milk consumed by the duration of the feed (minutes). 

### 2.6. Bradycardia and Desaturation Events

Bradycardia was defined as <90 bpm and desaturation episodes defined as <91% were recorded during each of the monitored feeds.

### 2.7. Statistical Analysis

Data analysis was run in R v3.0.3 for Mac OS X (Apple Inc., Cupertino, CA, USA), with additional packages nlme and lattice. Unless otherwise specified, summary statistics are presented as mean ± SD (range), while model parameters are presented as estimate (95% confidence interval (95% CI)). Model parameters are not presented for transformed data. 

Summary variables presented in Table 1 were compared using either paired *t*-tests or paired Wilcoxon signed rank tests. Comparison of infants included in this study with those omitted used either independent samples *t*-tests or Fisher’s Exact test. All other analyses used linear mixed effects models to investigate differences by feed type, and simultaneous linear regression to investigate individual patterns, using the same fixed effects as the final mixed effects model. Model fit was assessed with residual plots. Where more than one random effects grouping was considered, models were compared using likelihood ratio tests. Multiple comparisons used Tukey’s all pair comparisons.

Intra-oral vacuum data consisted of 1641 suck burst and 1607 pause records. Single sucks (102 breast, 193 NT) were omitted from vacuum analyses (other than mean pressure) as these measures cannot be made on single sucks. Five records (three breast, two NT) with a sucking rate ≥ 200 sucks/min were omitted as this indicates measurement artifacts. Missing data included tongue movement for two NT feeds; nipple diameters at 15 mm in 37 records, as insufficient nipple was drawn into the mouth to make the measurement and milk intakes for four feeds in three infants. 

### 2.8. Tongue Movement

Linear mixed effects models were used to investigate relationships between feed type and N-HSPJ, IOD, and nipple diameter after accounting for tongue position and measurement location. Main effect, two-way interaction involving feed, and three way interaction models investigated whether there was differing movement patterns between feed types. For each fixed effects model, four random intercept groupings were compared, allowing for differences between infants, amount of tongue movement between infants, and between feed types between infants, in the amount of tongue movement by feed type within the infant.

### 2.9. Intra-Oral Vacuum

All vacuum and sucking measures were analysed with linear mixed effects models. Minimal random effects considered were random intercepts by feed type within infant. Random slopes by time (linear or second order polynomial), burst type, and suck burst vacuum were also considered as random effects when included in the fixed effects; groupings were by infant or by feed within infant. Fixed effects are detailed for each analysis. Models, which did not converge, were considered to be misspecified, and alternate fixed and/or random effects were considered. Suck burst/pause duration and sucking events data were log transformed before analysis. Measurements of vacuum strength were square root transformed.

Effect of feed type on suck burst/pause durations considered both main effects of and interactions between feed type and burst type. Effect of feed type on changes over the course of the feed for suck bursts, for duration, sucking rate, and number of sucking events per burst, were analysed with either main effects or interaction models, with feed type and time since the beginning of the feed (linear or second order polynomial) as the fixed effects of interest. Effect of baseline and peak suck burst vacuum on overall sucking rate was analysed with either main effects or interaction models for feed type and vacuum. Number of sucks per burst was grouped as ‘single’ (1 suck), ‘immature’ (2–9 sucks), and ‘mature’ (10+ sucks). Number and proportion of suck bursts in the ‘single’ and ‘mature’ categories were compared using paired Wilcoxon signed rank tests.

Variability of vacuum within a feed was characterised by calculating the width of the inter-quartile range across all suck bursts for each of the pressures and compared with fixed effects of measure type and feed type, and random intercepts for feed type within infant.

### 2.10. Milk Intake

The association between milk intake and feed type was tested using a linear mixed effects model with random intercepts by infant and a fixed effect of feed type. Covariates are listed in Table 1, and vacuum variability measures; main effect and interactions with feed type were considered separately for each covariate. Milk intake data was square root transformed prior to analysis. 

## 3. Results

### 3.1. Participant Characteristics

Seventeen mothers and infants participated in this study. These infants were more likely than the others in the NT group of the larger study to be discharged home rather than back transferred (*p* = 0.002) and had spent longer on continuous positive airway pressure (CPAP) (*p* = 0.004).

Infant (11 female, 6 male; 8 singletons, 9 twins from 5 twin pairs) characteristics were birth gestation 29.2 ± 3.0 weeks (23.6–33.1 weeks), birth weight 1218 ± 43 g (540–1940 g), post-menstrual age (PMA) 33.6 ± 0.8 weeks (31.9–35.3 weeks)/post-natal age 4.4 ± 2.8 weeks (1.0–10.6 weeks) at introduction of full oral feeds, post-menstrual age 37.1 ± 1.2 weeks (35.9–40.1 weeks)/post-natal age 7.9 ± 3.6 weeks (2.7–16.6 weeks) at achievement of full oral feeds. Fifteen infants had been on respiratory support (CPAP: 3 infants for <1 week, 12 for longer; ventilation: 7 for <48 h, 4 for longer; oxygen: 13 for 1 h or longer).

### 3.2. Characteristics of Monitored Feeds

Characteristics of the monitored feeds are presented in Table 1. Monitored feeds occurred 2.7 ± 1.7 (0, 5.9) weeks after introduction of suck feeds and −0.8 ± 1.0 (−2.7, 1.1) weeks after achievement of full suck feeds. Nipple shields were used during 15/17 breastfeeds. 

### 3.3. Tongue Movement

Nipple diameters for the breastfeed and the NT are documented in Table 2.

Effect sizes (95% CI, associated *p*-value) for the main effects models and models with interactions involving feed type are presented in Table 3. No associations were seen between feed type and either N-HSPJ distance or intra-oral depth (*p* > 0.21). The amount of inferior movement (IOD) was between −2.4 ± 0.8 and 3.4 ± 0.8 mm greater during NT feeds. 

**Figure 1:**
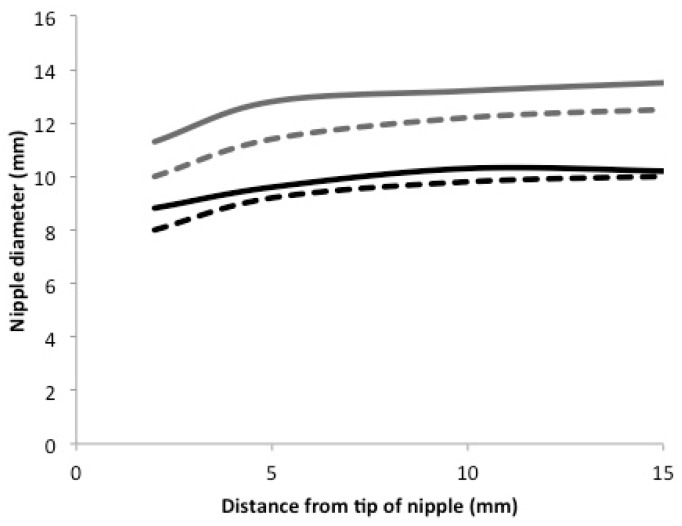
The movement of the infant tongue from its uppermost point to its lowest point for a breastfeed and feed with the novel teat. Solid grey line: breastfeed tongue down; dashed grey line: breastfeed tongue up; solid black line: novel teat tongue down; dashed black line: novel teat tongue up.

Nipple diameters were on average 2.3 mm (1.0, 3.6) smaller during NT feeds (Table 3; *p* = 0.002). The amount of change between tongue up and tongue down was on average 0.6 (−0.1, 1.3) mm greater during NT feeds. Overall, the pattern of nipple diameter measurements did not differ by feed, except at 2 mm from the tip of the nipple, where larger values were seen during NT feeds (Figure 1). The three-way interaction between measurement location, tongue position, and feed type was not significant (*p* = 0.80). Individual differences were seen in the relative patterns between NT and breastfeeds.

### 3.4. Intra-Oral Vacuum 

Summary statistics for intra-oral vacuum are presented in Table 4.

Changes in vacuum and sucking measures over the feed differed between infants (*p* < 0.001, all), with effects of feed on the pattern of change differing between infants (*p* < 0.001). Effect of feed type on burst (suck vs. pause) duration differed by infant (*p* < 0.001). Effect of suck burst vacuums on sucking rate differed by infant (*p* ≤ 0.001).

**Figure 2:**
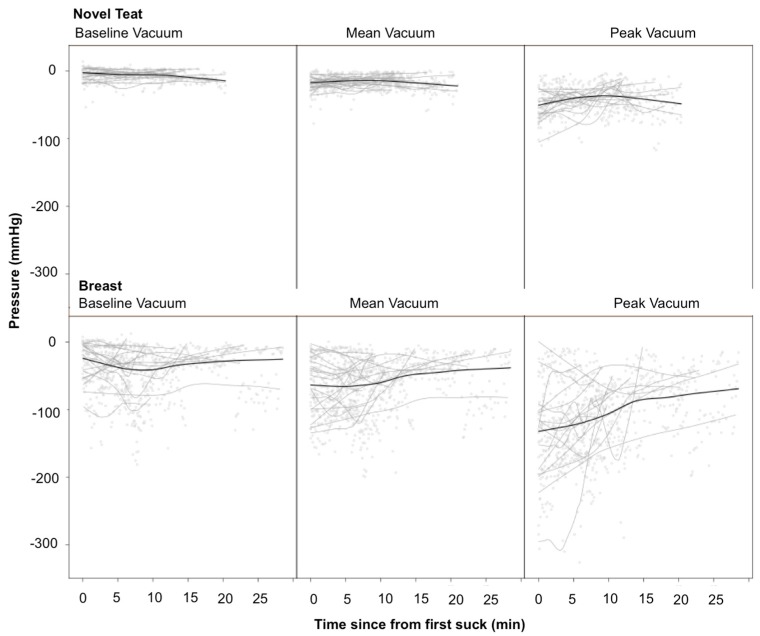
Sucking vacuums over the course of both the breastfeed and novel teat feed. Black lines are local regression smoothers for the full data set; grey lines are local regression smoothers indicating individual infant patterns.

Mean suck burst vacuums, mean pause vacuums, baseline suck burst vacuums, and peak suck burst vacuums were all stronger during breastfeeding (*p* < 0.001, *p* = 0.003, p < 0.001, p < 0.001, respectively). Mean suck burst vacuums varied over the course of breast (*p* = 0.011) but not NT (*p* = 0.68) feeds. Mean pause vacuums and peak suck burst vacuums did not display consistent patterns of change over the feed (*p* = 0.12, *p* = 0.22, respectively). Baseline suck burst vacuums showed consistent patterns over the feed, which differed by feed type (*p* = 0.043; Figure 2). Variability of all three measures was greater during breastfeeds (*p* < 0.008). Peak suck burst vacuums are more variable than baseline or mean suck burst vacuums (*p* < 0.001). The difference was smaller in NT feeds than breastfeeds but only significant for the comparison between peak and baseline suck burst vacuums (*p* = 0.021).

Suck bursts were shorter than pauses (*p* < 0.001), and no overall association with feed type was seen (*p* > 0.13). Suck burst durations did not change over the course of the feed (*p* = 0.29). At the beginning of the feeds, pause durations did not differ significantly by feed type (*p* = 0.063). Pause durations decreased over the course of NT feeds (*p* = 0.017), but no change was seen during breastfeeds (*p* = 0.37). Breastfeeds had more sucks per burst (*p* = 0.001) and higher sucking rates (10.4 (2.1, 18.6) sucks/minute, *p* = 0.001) than NT feeds; neither showed a consistent pattern of change over the feed (*p* = 0.32, *p* = 0.93, respectively). Higher sucking rates were associated with few sucks per burst (*p* = 0.004), but this association did not differ by feed type (*p* = 0.88). No association was seen between suck burst vacuums and sucking rate (peak, *p* > 0.54; baseline, *p* > 0.082).

Suck bursts of ≥10 sucks occurred during all breast and 15 of the 17 NT feeds; single sucks occurred during 15/17 of the breast and NT feeds. The number (*p* = 0.002) and proportion (*p* = 0.017) of suck bursts with ≥10 sucks were higher in breastfeeds 9 (6, 12) (22.2% (15.8, 31.0)) than NT feeds 3 (1, 7) (8.8% (3.3, 12.5)). The number (*p* = 0.031) and proportion (*p* = 0.008) of single sucks were higher during NT feeds: 6 (3, 8) (10.0% (6.7, 15.2)) than during breastfeeds; 10 (4, 14) (24.1% (15.8, 32.9)). Proportion of bursts with 2–9 sucks did not differ (*p* = 0.78), being 64.0% (58.8, 69.5) and 63.3% (56.2, 68.2) of NT and breastfeed suck bursts respectively.

### 3.5. Bradycardia and Desaturation Events

Desaturation events were reported for 4 infants for 6 of the 32 feeds. The breast:NT incidence for the four infants with desaturations was 3:3, 1:3, 0:1, and 1:0. Bradycardia events were recorded for three infants only during breastfeeding; one infant had two episodes, and two infants had one episode. Data was not recorded for two feeds.

### 3.6. Milk Intake

No measureable milk intake occurred for one breastfeed; three breastfeeds had milk intakes of 4 mL or less, whereas milk intake from the NT feeds was never this low. Milk intakes were larger during NT feeds (*p* = 0.003) and for longer feeds (*p* = 0.011), when a greater proportion of the feed was spent sucking (*p* = 0.023) and/or had a greater number of suck bursts (*p* = 0.007). The association between milk intake and feed type remained significant after accounting for each of the covariates *(p* < 0.004); no significant interactions were seen (*p* > 0.10). No association was seen between milk intake and any of the vacuum variability measures (*p* > 0.32).

## 4. Discussion

This study has found that movement of the tongue to create a vacuum with the NT was similar to that of breastfeeding. Whilst infants were required to exert a vacuum to remove milk from the NT, the intra-oral vacuums were weaker compared to the breast. Infants, however, consistently removed more milk from the NT than the breast.

### 4.1. Tongue Movement

The introduction of bottles during the establishment of breastfeeding in the term infant is considered detrimental due to the potential of nipple confusion, where the infant refuses the breast [28,29]. Whilst there is little evidence of nipple confusion, the infant has been shown to be highly adaptable. Infants will elect to use compression of the tongue to remove milk from a teat if it is easier than using vacuum. However, if the teat is designed to minimise or exclude the compression effect [30], the infant will employ vacuum to remove milk. Healthy preterm infants fed with an NT that only released milk with the application of vacuum moved their tongue in a similar fashion to breastfeeding (Figure 1). During a breast or NT feed, the infant drew their tongue downward, the nipple/NT expanded evenly, and intra-oral vacuum strength increased to peak vacuum (minimum pressure). Milk flowed into the cavity bounded by the hard and soft palate, the nipple, and the tongue surface. As the tongue was raised, intra-oral vacuum strength reduced to baseline vacuum (maximum pressure), milk was cleared under the soft palate to the phalangeal area and the nipple/NT diameter decreased (Figure 1). The only difference was that the tip of the NT was larger than the nipple (Figure 1) due to its shape and being less compressible than the human nipple. The absence of peristaltic tongue action with the NT compared to other teats [31,32] can be attributed greater thickness of silicone at the base of the NT, which minimised infant tongue compression. While the pattern of tongue movement was similar to breastfeeding, the degree of movement was greater during the breastfeed (Figure 1), which was likely due to the difference in elasticity between the nipple and the NT. Further, whilst most of the infants in this study used a nipple shield to feed, we have shown previously that tongue movement with a nipple shield is not different to tongue movement without [20].

Nipple shields are often used in our NICU to facilitate breastfeeding in preterm infants, enabling them to remain attached to the breast. Evidence shows that for the hospitalised preterm infant, nipple shield (NS) use is associated with improved milk removal [33]. There is limited evidence regarding the impact of NS on breastfeeding exclusivity and duration beyond discharge from hospital. The absence of a relationship between nipple shield use and age at achievement of exclusive breastfeeding [34] has been reported, while lower rates of exclusive breastfeeding have been reported in infants that have used a shield (49% with nipple shield use and 66% without) [35]. Indeed, several factors impact the achievement of exclusive breastfeeding in preterm infants [35]. Complicating factors such as low maternal milk production and/or low infant intraoral vacuum with subsequent insufficient milk transfer are rarely measured. Thus there is a critical need to understand the mechanisms by which nipple shields function to enhance transfer of milk from the breast in the context complicating maternal and infant factors.

The tongue movements of the preterm infants studied are also comparable to that documented in term breastfeeding infants [26,36,37,38] as well as a larger cohort of breastfeeding preterm infants also recruited from this study [20]. The absence of any marked peristaltic tongue action during breastfeeding or feeding with the NT is in contrast to other studies of bottle-feeding, implying the design of the teat influences tongue movement [31,39]. The unique stresses of tongue movement during feeding likely impact the development of the form and structure of facial structures, particularly the palate [40,41], evidenced by a 68% reduction of malocclusion if infants are breastfed [42]. Further, anthropological observations of prehistoric skulls show broader flatter palates suggesting the forces exerted during breastfeeding influences developing structures [43]. Given the malleability of preterm infants’ facial structure and immaturity of muscular control and strength, it could be potentially beneficial to emulate the tongue motion of breastfeeding in the event of the unavailability of the mother to breastfeed.

Positioning of the teat/nipple in the oral cavity is important in order to avoid gagging, which may progress to oral aversion and also to ensure optimal placement of the milk bolus so that it can be easy cleared in co-ordination with breathing and swallowing [44]. We found no difference in the positioning of the nipple and NT in relation to the N-HSPJ (Table 2), indicating good positioning [45]. The infants, however, drew their tongue down more (IOD) during the NT feed, which may be due to not having to apply as much vacuum to obtain a bolus. Thus, greater volumes of milk were transferred during the NT feeds.

### 4.2. Intra-Oral Vacuum

Preterm infants are recognised to have individual trajectories to attainment of feeding milestones that are complicated by immaturity and associated co-morbidities [28,46]. Commensurate with this notion, measures of preterm intra-oral vacuum were variable across the course of a feed, and patterns were different between individual infants in this study. 

One of the factors implicated in more effective and efficient bottle-feeding is increased strength of vacuum [47]. The breastfeeding preterm infants in this study displayed vacuums approximately a half to two-thirds that applied by term infants [36,48] (Table 4). This is consistent with our larger descriptive study of preterm breastfeed infants [20] and also the low milk transfer from the breast (Table 1). Peak vacuums were also weaker when infants fed at the NT likely due the threshold set for milk flow from the NT while peak vacuums at the breast are due to the greater flexibility of the human nipple. With the NT the infant may also adjust vacuums applied to modulate milk flow rate so that suck-swallow-breathe co-ordination is not compromised. 

To suck effectively, infants latch to the breast or the teat, and this is measured as the baseline vacuum. It is important to form a seal to the breast/teat to stretch and position the nipple/teat optimally in the oral cavity in relation to the N-HSPJ to remove milk [49]. If this is not possible, the infant may compensate by allowing milk to spill out of the mouth [50]. The baseline seal to the NT increased in strength over the feed and weakened during the breastfeed (Figure 2), perhaps reflecting fatigue due to the stronger applied vacuums. In contrast, term breastfed infants increase their baseline vacuums over a feed [49]. These changes might be due to changes in breast elasticity due to the drainage of milk, whereas preterm infants remove small volumes from the breast (Table 1). 

Mature sucking patterns are frequently described as the ability to extend suck bursts and reduce the lengths of pauses [21]. In contrast, our preterm infants exhibited suck bursts that were shorter than the pauses for both the breast and NT feeds. This apparent reversal suggests a lack of feeding maturity and is further reflected by low milk transfer along with the low proportion of suck bursts comprised of >10 sucks, which is also considered a marker of feed progression. Single sucks were also frequently identified in nearly all breast and NT feeds which is rarely seen during a term breastfeed and itself perhaps a gauge of immaturity of oral feeding. Interestingly, the frequency and proportion of ‘mature’ suck bursts was higher in breastfeeds (nine per feed; 22.2%) than NT feeds (three per feed; 8.8%), yet milk transfer was more effective in the NT feeds. 

Often, clinical observations of slowing of sucking rate are interpreted as a change from non-nutritive to nutritive sucking [17]. Infants in this study did not change their suck frequency across a feed but sucked more rapidly during breastfeeding. Conversely, sucking rates increase across a breastfeed in term infants [49]. This absence of change across a feed makes sucking rate an unreliable indicator of milk intake particularly during breastfeeding. Further, suck rate is not indicative of vacuum strength, with no relationships demonstrated with either peak or baseline vacuum. 

Little attention is paid to the length of time a preterm infant pauses at the breast/teat, and long pauses are considered to be due to fatigue or state. We found that pause durations decreased over the course of NT feeds whilst remaining constant during breastfeeds. This may be a congruent with better suck-swallow-breathe co-ordination as the NT feed progressed. Further studies identifying the nutritive and non-nutritive portions of the feed as well as measurement of swallowing and breathing would serve to clarify these differences. 

Cardiorespiratory stability is a major concern during oral feeding of the preterm infant. It is well documented that bottle-feeding is associated with more episodes of desaturation and bradycardias compared to breastfeeding [51]. Due to the design of the NT, we expected that the cardiorespiratory stability of the infant fed with the NT would be similar to breastfeeding due to the infant having the opportunity to self regulate their intake from the NT as they would from the breast. However, desaturation occurred overall in only six of 32 feeds (20%), and these were in only four infants. Similarly, bradycardia events were rare and occurred in three infants where one had two episodes and two had one episode during breastfeeding only. Although the numbers are too small to conduct meaningful statistical analysis, there did not appear to be more desaturations during NT feeds. 

Good growth is critical for the preterm infant as under nutrition is associated deficits in neurodevelopment and increased risk of sepsis and necrotizing enterocolitis [52]. Thus, the ingestion of adequate volumes of milk from every oral feed is imperative. We have demonstrated in a previous study that intakes range from 0 to 40 mL in preterm breastfed infants [18], which is consistent with Meier et al. [33], who showed intakes from 2–62 mL, and this study. Typically, infants are supplemented to achieve the prescribed volume but without objective measures of the volume of milk ingested at a breastfeed. Overall, infants received significantly greater volumes from the NT that were much closer to the prescribed volume by the neonatologist. As aforementioned, the small volumes of milk removed from the breast is likely due to the low strength of vacuum generated, as [36,49] (Figure 2) this has been associated positively associated with milk intake in term breastfed infants [53]. Longer breastfeeds and a greater proportion of the breastfeed spent sucking usually resulted in increased milk intake, which is in contrast to the NT, where any suck resulted in milk transfer. Clearly, more research is required to identify factors that would improve milk intakes from the breast.

Clinically encouragement of a sucking action similar to breastfeeding during bottle-feeding is likely to confer immediate advantages such as increased infant feed regulation without reduction in feed volume and potentially less nipple ‘confusion’. In the longer term, it may also impact development of the oro-facial development by reducing the incidence of conditions such as malocclusion, which is higher in preterm infants [54,55], and sleep disordered breathing where factors such as palate shape, hypotonia, and bottle-feeding appear to contribute [56].

The limitations of the study include the small numbers of participants that were not using a nipple shield to feed; thus, we were not able to directly compare breastfeeding without a shield and with the NT. A more extensive study is required to assess the sucking dynamics with and without a nipple shield. Further, these infants represent a relatively ‘healthy’ population that began oral feeding at approximately 34 to 35 weeks post menstrual age therefore the results are not applicable to younger infants or those with co-morbidities that are known to adversely affect feeding trajectories. It is anticipated that future studies will investigate suck, swallow, breathe co-ordination, and sucking during breastfeeding and feeding with the NT and conventional teat. 

## 5. Conclusions

The novel teat, which only released milk when the preterm infant applied vacuum, encouraged a similar tongue action to breastfeeding and infants transferred a greater volume of milk compared to breastfeeding. Intra-oral vacuums were lower in strength with the novel teat compared to the breast. Further research is required to compare suck-swallow-breathe patterns with the novel teat and breastfeeding and improve transfer volumes at the breast. 

## Figures and Tables

**Table 1:** Infant and monitored feed characteristics for breastfeeding and the novel teat.

Infant and Feed Characteristics	Breast	Novel Teat	Difference	*p*-Value
Feed duration (min)	10.6 (9.0, 20.3)	12.3 (9.1, 16.6)	1.9 (−4.0, 9.4)	0.28
Time sucking (min)	3.4 (2.4, 7.8)	2.7 (1.8, 3.7)	1.4 (0.0, 4.6)	0.027
Time Pausing (min)	7.9 (5.2, 11.7)	9.5 (5.8, 11.4)	0.3 (−3.6, 2.4)	0.078
Mean vacuum (mmHg)	−30.7 (−50.0, −21.2)	−6.0 (−14.0, −3.4)	−24.6 (−46.4, −9.2)	<0.001
Proportion of feed spent sucking	0.36 ± 0.16	0.27 ± 0.15	0.14 (−0.07, 0.09)	0.072
Number of suck bursts	35 (29, 66)	34 (25, 51)	6 (−5, 29)	0.28
Prescribed volume (mL)	46.1 ± 8.2	45.7 ± 7.1	0 (0, 0)	0.36
Milk intake (mL)	12 (3, 15.5)	33 (22.5, 42.5)	−19 (−35, −4)	0.007
Milk transfer (mL/min)	2.2 (1.2, 3.2)	9.2 (7.0, 13.2)	−7.9 (−12.6, −3.4)	<0.001
Age at monitored feed PMA (weeks)	36.3 ± 1.8	36.4 ± 1.5	−0.14 (−0.3, 0)	0.74
Post-natal (weeks)	7.1 ± 4.2	7.2 ± 3.9		
Weight at monitored feed (g)	2120 ± 421.7	2146 ± 294.8	−65 (−129, 0)	0.50

**Table 2:** Measures of ultrasound images during breastfeeding and feeding with the novel teat. N-HSPJ: nipple-hard soft palate junction.

Infant Intra-Oral and Nipple Diameter Measures	Breast		Novel Teat	
	Tongue Up	Tongue Down	Tongue Up	Tongue Down
N-HSPJ distance (mm)	7.1 ± 2.9	5.2 ± 2.6	5.6 ± 1.4	4.7 ± 1.4
Intra-oral depth (mm)	0.3 ± 0.5	4.2 ± 2.0	0.1 ± 0.2	4.1 ± 1.3
Nipple diameters (mm)				
2 mm	10.0 ± 3.0	11.3 ± 2.3	8.0 ± 1.1	8.8 ± 1.1
5 mm	11.4 ± 2.6	12.8 ± 2.5	9.2 ± 0.9	9.6 ± 1.3
10 mm	12.2 ± 2.5	13.2 ± 2.4	9.8 ± 1.0	10.3 ± 1.3
15 mm	12.5 ± 2.7	13.5 ± 2.6	10.0 ± 1.0	10.2 ± 1.5

**Table 3:** Effect (parameter ± SE) of tongue position (tongue down) and feed type (NT) on N-HSPJ distance, and intra oral depth; and tongue position, feed type, and measurement location (location) on nipple diameters. Reference levels are tongue position is up, feed type is breastfeed, and location is 5 mm from the nipple tip. Random effects indicates the sub-groups of the data for which random intercepts were fitted, allowing for individual (infant) differences between measurements. The main effects models include all considered terms; interaction models consider only interactions, which include feed type.

Random Effects	N-HSPJ		IOD		Nipple Diameters	
	Coeff (95% CI)	*p*-Value	Coeff (95% CI)	*p*-Value	Coeff (95% CI)	*p*-Value
	Feed within Infant		Tongue Position within Feed within Infant		Tongue Position within Feed within Infant	
Main effects models						
Reference ^a^	5.2 (4.1, 6.2)	-	0.3 (−0.2, 0.7)	0.30	11.2 (10.3, 12.2)	-
Tongue down	1.6 (1.3, 1.8)	<0.001	3.9 (3.4, 4.5)	<0.001	1.0 (0.7, 1.4)	<0.001
Novel Teat	−0.5 (−1.9, 0.8)	0.42	−0.1 (−0.7, 0.5)	0.72	−2.3 (−3.6, −1.0)	0.002
Location						<0.001 ^b^
2 mm	-	-	-	-	−1.2 (−1.4, −1.0)	<0.001
10 mm	-	-	-	-	0.6 (0.4, 0.8)	<0.001
15 mm	-	-	-	-	0.8 (0.6, 1.0)	<0.001
Interaction models						
Reference ^a^	5.1 (4.0, 6.1)	-	0.3 (−0.5, 0.8)	0.28	11.1 (10.1, 12.1)	
Tongue down	1.7 (1.3, 2.1)	<0.001	3.8 (3.1, 4.6)	<0.001	1.3 (0.9, 1.8)	<0.0001
Novel Teat feed	−0.4 (−1.8, 1.1)	0.60	−0.2 (−1.0, 0.6)	0.61	−2.0 (−3.4, −0.6)	0.007
Location						<0.0001 ^b^
2	-	-	-	-	−1.4 (−1.7, −1.1)	<0.0001
10	-	-	-	-	0.6 (0.3, 0.9)	<0.0001
15	-	-	-	-	0.9 (0.6, 1.2)	<0.0001
Tongue down Teat	−0.4 (−0.9, 0.2)	0.21	0.2 (−0.9, 1.3)	0.72	−0.6 (−1.3, 0.1)	0.069
Location Teat						0.010 ^b^
2 mm	-	-	-	-	0.4 (0.002, 0.8)	0.039
10 mm	-	-	-	-	−0.1 (−0.5, 0.3)	0.56
15 mm	-	-	-	-	0.3 (−0.7, 0.2)	0.26

^a^ *p*-Values not included for reference levels except models for IOD, as the question ‘is this significantly different from zero’ is not meaningful for other analyses. ^b^ Omnibus *p*-values, indicating the overall chance that there is at least measurement points with significantly different measurements. *p*-Values for each of the measurement locations show significance of difference with respect to measurement made at 5 mm from nipple tip.

**Table 4:** Summary statistics for infant intra-oral vacuum measures, separated by feed and burst type. Data is presented as median (IQR) (range).

	Breast		Novel Teat	
	Sucks (*n* = 912)	Pauses (*n* = 895)	Sucks (*n* = 729)	Pauses (*n* = 712)
Duration * (s)	4.5 (2.7, 7.4)	5.4 (2.7, 9.0)	2.4 (1.3, 4.6)	5.2 (2.4, 11.6)
	(0.5, 52.4)	(0.5, 712.6)	(0.4, 132.8)	(0.6, 379.7)
Sucking Events * (*n*)	6 (3.8, 9)	-	3 (1, 5)	-
	(1, 54)		(1, 147)	
Sucking Rate (*n*/min)	88.0 (73.7, 102.6)	-	77.6 (62.4, 96.6)	-
	(19.0, 194.4)		(14.2, 191.2)	
Vacuum (mmHg)				
Baseline	−31.1 (−60.0, −12.7)	-	−5.8 (−11.0, 0.1)	-
	(−181.0, 12.4)		(−53.3, 14.3)	
Mean *	−53.6 (−89.3, −31.5)	−23.9 (−48.8, −11.7)	−15.5 (−21.8, −8.9)	−5.1 (−11.0, −0.6)
	(−199.3, 0.4)	(−185.1, 1.9)	(−77.6, 2.2)	(−79.6, 5.3)
Peak	−106.2 (−153.0, −65.5)	-	−40.0 (−54.6, −27.1)	-
	(−325.7, −9.6)		(−116.7, −3.1)	
Vacuum Variability				
Baseline	23.4 (17.3, 37.0)	-	5.5 (4.4, 6.1)	-
	(5.3, 47.3)		(1.4, 12.0)	
Mean *	26.2 (25.0, 41.6)	-	9.3 (7.8, 10.5)	-
	(11.2, 73.9)		(3.6, 18.9)	
Peak	50.0 (33.0, 60.7)	-	25.4 (20.6, 29.3)	-
	(18.9, 154.3)		(3.9, 39.4)	

* includes single sucks.
